# Draft Genome Sequences of Two Pseudomonas aeruginosa Multidrug-Resistant Clinical Isolates, PAL0.1 and PAL1.1

**DOI:** 10.1128/MRA.00940-18

**Published:** 2018-11-01

**Authors:** Teddy Grandjean, Rémi Le Guern, Claire Duployez, Karine Faure, Eric Kipnis, Rodrigue Dessein

**Affiliations:** a Host-Pathogens Translational Research Group, Faculty of Medicine of Lille, University of Lille Nord de France, Lille, France; b Institut de Microbiologie, CHU Lille, Lille, France; c Unité de Maladies Infectieuses, CHU Lille, Lille, France; d Service de Réanimation Chirurgicale, CHU Lille, Lille, France; Georgia Institute of Technology

## Abstract

Pseudomonas aeruginosa infections are challenging due to intrinsic and acquired resistance mechanisms. We report here the draft genome sequences of two multidrug-resistant strains—PAL0.1, isolated from the airways of an intensive care unit (ICU) patient with ventilator-associated pneumonia, and PAL1.1, isolated from blood cultures of an ICU patient with sepsis.

## ANNOUNCEMENT

Pseudomonas aeruginosa is one of the most common nosocomial pathogens, responsible for acute infections such as ventilator-associated pneumonia and infections in immunocompromised hosts, such as burn patients. Due to increasing multidrug resistance (MDR), P. aeruginosa is among the top-priority pathogens according to the World Health Organization (1). In addition to intrinsic and acquired antibiotic resistance mechanisms (decreased outer membrane permeability, enzymatic antibiotic deactivation, and efflux pumps), P. aeruginosa forms biofilms that decrease exposure to antimicrobials. Treating MDR P. aeruginosa infections is increasingly challenging and requires new therapeutic approaches. Two representative MDR clinical strains related to the well-described PAO1 and PA14 phylogenetic clades were isolated from patients in the Lille University Teaching Hospital (CHU Lille, France)—PAL1.1, isolated from the airways of an intensive care unit (ICU) patient with ventilator-associated pneumonia, and PAL0.1, isolated from blood cultures of an ICU patient with sepsis. Both strains were isolated from blood cultures using the Virtuo automated system (bioMérieux, Marcy-l'Étoile, France) and were identified by matrix-assisted laser desorption ionization–time of flight (MALDI-TOF) mass spectrometry (Bruker Daltonics, Inc., Billerica, MA). The complete genome sequences of these strains were determined in order to assess lectin-specific bacterial adhesion inhibiting glycosylated calixarenes against multidrug-resistant (MDR) Pseudomonas aeruginosa strains.

Genomic DNA from P. aeruginosa strains PAL0.1 and PAL1.1 was extracted after an overnight culture using a QIAamp DNA minikit (Qiagen, Hilden, Germany). Libraries were prepared using the Nextera XT DNA Library prep kit (Illumina, San Diego, CA), followed by paired-end (2 × 150-bp) sequencing on an Illumina HiSeq platform. Genome coverage was about 650× for each isolate. Paired reads were filtered, and Nextera adapters were removed using Trimmomatic (2). Processed reads were *de novo* assembled with the Unicycler pipeline on a Galaxy server version 0.4.1.1 using default settings (3). Annotation was performed using the NCBI Prokaryotic Genome Annotation Pipeline version 4.5. The genome lengths are 7,040,354 bp and 6,780,687 bp with G+C contents of 65.87% and 66.04% for PAL0.1 and PAL1.1, respectively. The assembly reported 131 contigs with an *N*_50_ value of 202,317 bp for PAL0.1 and 108 contigs with an *N*_50_ value of 222,705 bp for PAL1.1.

The PAL0.1 genome annotation consists of 6,736 coding sequences (CDSs) and 65 structural RNAs. Detection of acquired antimicrobial resistance genes using ResFinder version 3.0 (4) found 2 aminoglycoside resistance genes [*aacA29* and *aph(3′)-llb*], 3 beta-lactam resistance genes (*blaOXA-50*, *blaPAO*, and *blaVIM-2*), the fosfomycin resistance gene *fosA*, and the sulfonamide resistance gene *sul1*.

The PAL1.1 genome annotation consists of 6,479 CDSs and 68 structural RNAs. Detection of acquired antimicrobial resistance genes using ResFinder version 3.0 found 3 aminoglycoside resistance genes [*aph(3′)-llb*, *ant(2ʺ)-la*, and *aadA11*], 2 beta-lactam resistance genes (*blaOXA-50* and *blaPAO*), the fosfomycin resistance gene *fosA*, the sulfonamide resistance gene *sul1*, the phenicol resistance gene *catB7*, and the trimethoprim resistance gene *dfrB1*.

Multilocus sequence typing (MLST) was performed using MLST version 2.0 from the Center for Genomic Epidemiology (5). PAL0.1 (PAO1 clade) is a Verona integrin-encoded metallo-beta-lactamase (VIM-2)-producing strain that belongs to the sequence type 111 (ST-111), a clonal strain responsible for nosocomial infection epidemics worldwide (6, 7). PAL1.1 (PA14 clade) shares an acquired resistance genotype commonly observed worldwide among clinical P. aeruginosa strains (8) belonging to ST-2613.

### Data availability.

The whole-genome shotgun project of PAL0.1 and PAL1.1 has been deposited in DDBJ/EMBL/GenBank under the accession no. QFRL00000000 and QFRM00000000, respectively. The SRA accession no. is SRP155966.
